# Association of Systemic Inflammation Response Index With All‐Cause and Cardiovascular Mortality Among Individuals With Depression: NHANES 2005–2018

**DOI:** 10.1002/brb3.71437

**Published:** 2026-04-22

**Authors:** Ying Tang, Songfeng Zhao, Qiuping Huang, Xinxin Chen, Shuhong Lin, Yifan Li, Jingyue Hao, Zhenjiang Liao, Hongxian Shen

**Affiliations:** ^1^ Department of Psychiatry National Clinical Research Center for Mental Disorders Changsha Hunan China; ^2^ Department of Neurosurgery the Third Xiangya Hospital of Central South University Changsha Hunan China; ^3^ Department of Psychology School of Humanities and Management Hunan University of Chinese Medicine Changsha Hunan China

**Keywords:** all‐cause mortality, cardiovascular mortality, depression, systemic inflammation response index

## Abstract

**Background:**

The Systemic Inflammation Response Index (SIRI) has recently been investigated as a new marker of inflammation. This study investigated the association between SIRI and all‐cause and cardiovascular mortality risks in patients with depression.

**Methods:**

Based on data from the National Health and Nutrition Examination Survey (NHANES), 2410 participants with depression were included and followed up for all‐cause and cardiovascular‐related deaths through December 31, 2019. Depression was identified as a 9‐item Patient Health Questionnaire (PHQ‐9) score ≥10. Weighted multivariate Cox proportional hazards regression analyses assessed the relationship between SIRI and all‐cause and cardiovascular mortality. Time‐dependent receiver operating characteristic (ROC) curve analysis evaluated SIRI's accuracy in predicting survival outcomes.

**Results:**

Among the 2410 participants, 282 all‐cause death events and 84 cardiovascular disease (CVD)‐related death events were documented. In the fully adjusted models, the highest SIRI tertile was significantly associated with increased risk of all‐cause and cardiovascular mortality with hazard ratios (HRs) (95% CIs) of 1.26 (0.79, 2.02) for all‐cause mortality and 2.50 (1.22, 5.13) for cardiovascular mortality. This association remained consistent in subgroup and sensitivity analyses. The time‐dependent ROC curve showed that the areas under the curve of the 3‐, 5‐, and 10‐year survival rates were 0.702, 0.692, and 0.644 for all‐cause mortality, and 0.685, 0.714, and 0.653 for cardiovascular mortality, respectively.

**Conclusion:**

An elevated SIRI is associated with an increased risk of all‐cause and cardiovascular mortality among patients with depression.

## Graphical abstract

In 2410 NHANES adults with depression, the highest SIRI tertile (HR 1.26 all‐cause; 2.50 cardiovascular) predicted greater mortality; time‐dependent AUCs (0.70–0.64) validate SIRI as a low‐cost blood biomarker for long‐term risk stratification.

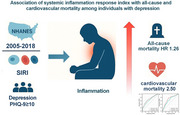

## 1 Introduction

Depression, a common mental health disorder, severely impairs patients' social functions, diminishes their quality of life, and places a heavy burden on individuals, families, and society (Malhi and Mann 2018; Sousa et al. 2022). Estimates of depression's lifetime prevalence differ widely across countries due to factors like diagnostic tools and statistical methods, yet cross‐country data consistently documents a significant prevalence. With an overall lifetime risk of about 15–18%, depression impacts a large global population, affecting nearly one in five people at some point in their lives (Bromet et al. 2011; Kessler and Bromet 2013; Malhi and Mann 2018). Patients with depression have been shown to have higher mortality, contributing to a shortened life expectancy (Cuijpers and Smit 2002). People with depression generally live 7.9 years less than their healthy counterparts (Pratt et al. 2016). Depression is currently diagnosed based on symptoms that can be assessed using the Patient Health Questionnaire, Beck Depression Inventory, Hamilton Depression Rating Scale, Center for Epidemiologic Studies Depression Scale, or Zung Self‐Rating Depression Scale (Tommasi et al. 2018). However, symptoms alone are insufficient to guide precise decisions regarding drug use or long‐term prognosis.

Several studies have explored the pathogenesis and identification of depression (Levis et al. 2019). A growing body of research is using noninvasive neuroimaging tools to explore changes in brain structure and function among individuals with depression. Studies using brain magnetic resonance imaging (MRI) have shown that patients with depression exhibit abnormal patterns of connectivity in the default mode network compared with healthy controls, which has been linked to self‐referential processing, rumination, and emotion regulation (Liston et al. 2014; Yun and Kim 2021). Although significant advances have been made in understanding the pathogenesis of and treatment depression, its etiology is shaped by   multiple genetic and environmental factors, and substantial heterogeneity across studies makes findings difficult to replicate; therefore, current evidence remains insufficient to fully explain the etiology of depression(Guideline Development Panel for the Treatment of Depressive Disorders 2022; Malhi and Mann 2018). Additionally, some patients do not respond well to existing treatments, and some patients with symptom improvement develop residual symptoms that continue to negatively affect their functioning and increase the risk of recurrence (Yun and Kim 2021; Zhang et al. 2018). Thus, the pathophysiological mechanisms underlying depression require further investigation. It is important to identify effective, inexpensive, and readily available biomarkers for treatment.

Numerous studies have identified a connection between depression and inflammation, suggesting that inflammatory processes play a role in the onset and persistence of depression, leading to the proposal of the “inflammation hypothesis” (Kopschina Feltes et al. 2017; Osimo et al. 2019). Significantly, chronic inflammation can cause dysfunction in serotonin, norepinephrine, and dopamine systems (Song and Wang 2011). Individuals with depression have abnormal levels of inflammatory markers, immune cell numbers, and antibody titers in their blood (Beurel et al. 2020; Müller 2014). Multiple meta‐analyses have now confirmed highly consistent increases in blood levels of cytokines and inflammatory markers, including IL‐6, TNF‐α, IL‐10, and C‐reactive protein (CRP), in people with depression compared with healthy controls (Mac Giollabhui et al. 2020; Osimo et al. 2019). Moreover, CRP and TNF are associated with the severity of depressive symptoms, and studies have shown that changes in peripheral cytokine levels are associated with antidepressant treatment outcomes, indicating that inflammatory markers such as IL‐6 or CRP show promise as predictors of treatment response‐resistant depression (Song and Wang 2011). Presently, blood biomarkers are among the most commonly used research methods. However, many biomarkers are difficult to obtain or expensive to extract, limiting their large‐scale application. Indicators such as neutrophils, monocytes, lymphocytes, and platelets reflect the level of inflammation in the body and are cheap and easy to obtain. Recently, Systemic Inflammation Response Index (SIRI), a novel biomarker that integrates the peripheral counts of neutrophils, monocytes, and lymphocytes, has been shown to play an important role in stroke, autoimmune rheumatic diseases, and cardiovascular diseases (Xia et al. 2023; Xu et al. 2022; Zhang et al. 2021). As there has been little research on depression, this study aimed to determine the association between SIRI and all‐cause and cardiovascular disease (CVD mortality among patients with depression in the United States.

## 2 Method

### 2.1 Study Population

Data were obtained from the National Health and Nutrition Examination Survey (NHANES) 2005–2018, a nationally representative program that assesses the health and nutritional status of the US non‐institutionalized population. Data collection methods included household interviews, physical examinations at mobile centers, and laboratory tests. Details of the NHANES database are available at https://wwwn.cdc.gov/nchs/nhanes/.

The study protocol was approved by the Ethics Review Board of the National Center for Health Statistics. Written informed consent was obtained from all participants. This STROBE‐compliant cohort study initially identified 70,190 participants from NHANES 2005–2018.

The inclusion criteria for our study were (1) age ≥20 years; (2) complete data on Patient Health Questionnaire (PHQ‐9) score; (3) complete data on survival status; and (4) complete data on blood cells for SIRI calculation. Exclusion criteria comprised: (1) missing PHQ‐9 scores (*n* = 5485); (2) undocumented survival status (*n* = 54); (3) incomplete blood cell counts (*n* = 1366); (4) abnormal white blood cell counts (<4000 cells/µl or >10,000 cells/µl) (*n* = 4017); and (5) participants without depression (PHQ‐9 < 10, *n* = 26,417). After excluding 30,441 participants aged <20 years and applying other exclusion criteria, the final cohort included 2410 eligible individuals. A detailed selection flowchart is provided in Supplementary Figure S1.

### 2.2 Ascertainment of Mortality

Through December 31, 2019, survival status was determined by linking data to the National Death Index (NDI). According to the International Statistical Classification of Diseases, 10th Revision (ICD‐10) codes, CVD mortality was defined as codes I00‐I09, I11, I13, I20‐I51, and I60‐I69.

### 2.3 Definition of Depression

Depressive symptoms were assessed using the PHQ‐9, which contains nine items (Kroenke et al. 2001). Item scores range from 0 to 3, and the total score ranges from 0 to 27. The PHQ‐9 threshold of ≥10 was selected based on established clinical guidelines indicating moderate‐to‐severe depressive symptoms (Manea et al. 2015). Participants meeting this threshold were retained for depression‐related analyses.

### 2.4 Calculation of SIRI

Whole blood samples were collected from mobile examination centers and measured using a Beckman Coulter DxH 800 instrument. Neutrophil, monocyte, and lymphocyte counts were extracted and shown in 1000 cells/ul for calculation. SIRI was calculated as neutrophil count × monocyte count/lymphocyte count, as previously described (Qi et al. 2016).

### 2.5 Covariates

Covariates were selected based on previous epidemiological literature on inflammation and mortality as well as established cardiovascular risk factors (Chen et al. 2024; Lai et al. 2025). The continuous covariates included age, family‐to‐poverty ratio, and body mass index (BMI). Categorical covariates included sex (male/female), race (non‐Hispanic Black, non‐Hispanic white, other races), education (< high school, high school, > high school), smoking status (current, former, and never), drinking status (current, former, and never), hypertension (yes/no), diabetes mellitus (yes/no), and stroke history (yes/no).

### 2.6 Statistical Analysis

Because NHANES adopts a complex multistage probability sampling design, all analyses incorporated sampling weights to produce nationally representative estimates. Therefore, weighted t‐tests and weighted χ^2^ tests were used to compare baseline characteristics. The results were reported as means (standard error [SE]) for continuous variables and percentages (95% confidence interval [CI]) for categorical variables. We used a weighted 5‐knots restricted cubic spline (RCS) to explore the relationship between SIRI and mortality. Nonlinearity was assessed using the likelihood ratio test. To evaluate the association between SIRI and severity of depression, we conducted a comparative analysis of weighted mean SIRI scores across different levels of depression severity. Depression severity was categorized according to the standard PHQ‐9 classification (0–4: none or minimal depression; 5–9: mild depression; 10–14: moderate depression; 15–19: moderately severe depression; and 20–27: severe depression), as previously described by Kroenke et al. (2001).

We constructed Kaplan‐Meier survival curves with a risk table to show the difference in survival probabilities among different SIRI tertiles. Weighted multivariate Cox proportional hazards regression analyses were performed to evaluate the association between SIRI and all‐cause and CVD mortality, and the results were reported as hazard ratios (HRs) and 95% CIs. Three models were constructed to evaluate the association between SIRI and mortality outcomes. Covariates were selected based on previous epidemiological literature and their potential roles as confounding factors associated with inflammation and mortality (Xu et al. 2024). Model 1 was an unadjusted model. Model 2 was adjusted for demographic variables, including age, sex, and race. Model 3 was further adjusted for socioeconomic factors (education level and family‐to‐poverty ratio), lifestyle factors (smoking status and drinking status), and comorbidities (BMI, hypertension, diabetes mellitus, and history of stroke).

In addition, to illustrate the predictive ability of SIRI at different follow‐up durations, we carried out receiver operating characteristic (ROC) analysis with the R package “timeROC” (version 1.5). The predictive value was illustrated by the area under the curve (AUC).

We performed a subgroup analysis of potential confounding factors to estimate the effect of SIRI on different subgroups. Age was categorized as < 60 years and ≥ 60 years to distinguish middle‐aged adults from older adults, a cutoff commonly used in epidemiological and cardiovascular research to assess age‐related differences in disease risk and mortality. BMI was coded as obese (≥ 30 kg/m^2^) and nonobese (< 30 kg/m^2^). Interaction tests were cast between SIRI and different covariates by including a multiplicative term in the Cox regression model.

We further conducted a series of sensitivity analyses to test the robustness of the results and to assess potential bias. First, we excluded participants who died within 2 years of follow‐up to reduce the potential impact of reverse causation. Second, we further adjusted for the use of antidepressant medications in Model 4 to examine whether treatment status might influence the observed associations. Third, SIRI was categorized into quartiles instead of tertiles to assess whether the results were sensitive to the method of categorization. Fourth, the analyses were repeated among participants without depression to explore whether the association between SIRI and mortality differed by depression status. Fifth, we further evaluated the association between SIRI and depression.

The weighted statistical analyses were conducted using the R package “survey” (version 4.1) following the instruction of CDC analytical guidance. The missing data on covariates accounts for less than 10% and was substituted by the random forest algorithm with R package “missForest” (version 1.5) (Stekhoven and Bühlmann 2012). All statistical analyses were performed using R software (version 3.6.1). Statistical significance was defined as a two‐tailed *p*‐value < 0.05.

## 3 Results

Among the 2410 participants, 282 all‐cause death events and 84 cardiovascular‐related death events were documented. The median follow‐up period was 86 months. Table 1 shows the weighted baseline characteristics of depressive participants according to SIRI tertiles (T1: 0.00–0.79; T2: 0.79–1.30; T3: 1.30–6.40). Participants with higher SIRI tertiles tended to be men with higher incomes and educational levels. Additionally, the higher SIRI groups had an increased prevalence of morbidities such as hypertension, diabetes, and stroke. Compared with T1, the all‐cause and cardiovascular mortality rates of participants in the highest SIRI tertiles increased from 7.00% and 2.72% to 12.50% and 4.17%, respectively.

**TABLE 1:** Weighted characteristics of participants according to SIRI tertile.

	Overall	T1	T2	T3	*p*‐value
**Age (years)**	47.60 (0.45)	44.96 (0.68)	46.73 (0.65)	50.85 (0.78)	< 0.001
**Sex (%)**					0.003
Female	63.42 (0.03)	70.04 (2.22)	62.48 (2.54)	58.45 (2.09)	
Male	36.58 (0.02)	29.96 (2.22)	37.52 (2.54)	41.55 (2.09)	
**Race (%)**					< 0.001
Non‐Hispanic black	13.60 (0.01)	21.80 (1.99)	10.88 (1.11)	9.03 (0.87)	
Non‐Hispanic white	63.45 (0.03)	52.01 (3.11)	64.23 (2.36)	72.89 (1.90)	
Other races	22.95 (0.01)	26.20 (2.40)	24.89 (2.20)	18.07 (1.58)	
**BMI (kg/m^2^)**	30.24 (0.22)	29.96 (0.36)	29.84 (0.38)	30.92 (0.38)	0.090
**PIR**	2.16 (0.06)	2.04 (0.07)	2.14 (0.09)	2.29 (0.08)	0.030
**Education (%)**					0.010
< High school	24.96 (0.01)	27.14 (2.21)	23.45 (1.83)	24.53 (1.74)	
High school	26.45 (0.02)	30.75 (2.40)	25.76 (1.87)	23.29 (1.91)	
> High school	48.60 (0.02)	42.11 (2.17)	50.79 (2.17)	52.17 (2.65)	
**Smoking status (%)**					0.010
Never	40.51 (0.02)	43.93 (2.53)	43.84 (2.50)	34.05 (2.36)	
Former	24.23 (0.02)	21.09 (2.17)	22.73 (1.89)	28.57 (2.19)	
Current	35.26 (0.02)	34.98 (1.87)	33.43 (2.53)	37.38 (2.48)	
**Drinking status (%)**					0.250
Never	13.36 (0.01)	14.06 (1.41)	14.30 (1.43)	11.78 (1.20)	
Former	18.52 (0.01)	16.17 (1.93)	18.14 (1.87)	21.00 (1.67)	
Current	68.12 (0.03)	69.77 (2.29)	67.57 (2.26)	67.21 (1.93)	
**Hypertension**					< 0.001
No	53.07 (0.02)	57.64 (2.21)	56.25 (2.49)	45.76 (2.40)	
Yes	46.93 (0.02)	42.36 (2.21)	43.75 (2.49)	54.24 (2.40)	
**Diabetes mellitus**					< 0.001
No	79.67 (0.03)	84.26 (1.41)	81.30 (1.77)	73.92 (1.95)	
Yes	20.33 (0.01)	15.74 (1.41)	18.70 (1.77)	26.08 (1.95)	
**Stroke history**					0.100
No	93.10 (0.03)	94.09 (1.04)	94.21 (0.68)	91.08 (1.69)	
Yes	6.90 (0.01)	5.91 (1.04)	5.79 (0.68)	8.92 (1.69)	
**All‐cause death**					0.003
No	90.59 (0.03)	93.00 (1.13)	91.52 (1.13)	87.50 (1.33)	
Yes	9.41 (0.01)	7.00 (1.13)	8.48 (1.13)	12.50 (1.33)	
**CVD related death**					0.002
No	97.28 (0.03)	98.84 (0.40)	97.34 (0.60)	95.83 (0.73)	
Yes	2.72 (0.00)	1.16 (0.40)	2.66 (0.60)	4.17 (0.73)	

Abbreviations: BMI, body mass index; CVD, cardiovascular disease; PIR, poverty to income ratio.

**FIGURE 1:**
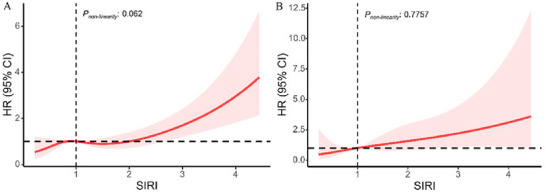
Association between SIRI and all‐cause mortality (A) and cardiovascular mortality (B) using a 5‐knot restricted cubic spline curve after adjusting all covariates.

**FIGURE 2:**
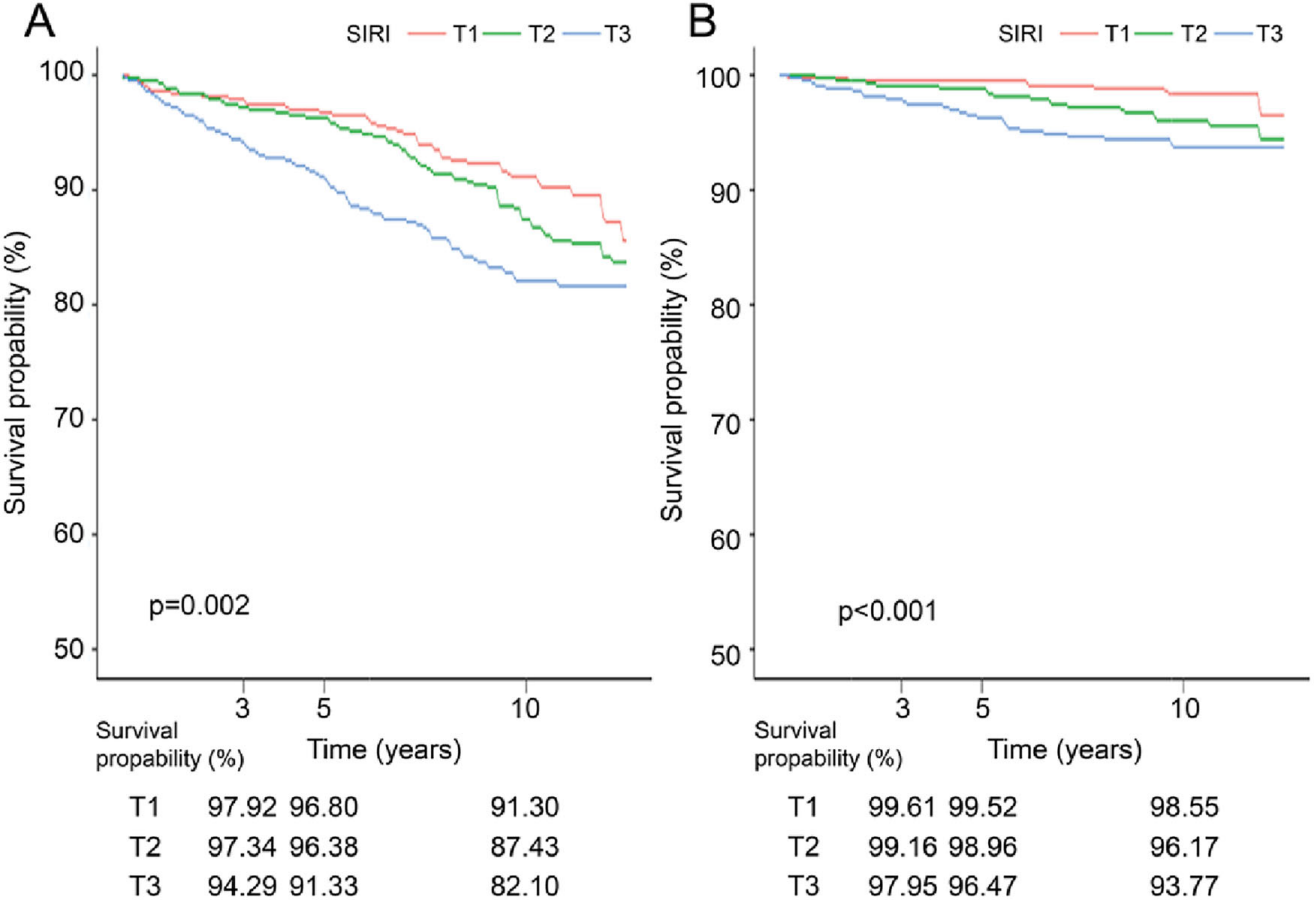
Kaplan–Meier curve and risk table for survival probabilities from all‐cause mortality (A) and cardiovascular mortality (B) of patients with depression.

Linear relationships between SIRI and all‐cause and cardiovascular mortality were identified among patients with depression (*p* > 0.05 for nonlinearities), as shown in Figure 1. The RCS curve indicated that all‐cause and cardiovascular mortality were positively associated with SIRI scores. In addition, the Kaplan–Meier curves and risk tables demonstrated that higher SIRI quantiles were associated with lower survival rates (Figure 2).

Participants with depression tended to have higher levels of SIRI scores, but the results were not statistically significant across the different levels of depression severity (Supplementary Figure S2).

Table 2 illustrates the results of the weighted Cox proportional hazards regression analyses. In the crude model, a unit increase in SIRI was associated with a 52% increase in all‐cause mortality and a 63% increase in cardiovascular mortality. In the fully adjusted models, a higher SIRI remained significantly associated with an increased risk of all‐cause and cardiovascular mortality. HRs (95% CIs) for the second and the third tertiles were 1.09 (0.72, 1.66) and 1.26 (0.79, 2.02) for all‐cause mortality and 2.16 (1.09, 4.30) and 2.50 (1.22, 5.13) for cardiovascular mortality, respectively.

**TABLE 2:** Association between SIRI and risks of all‐cause and cardiovascular mortality based on weighted Cox regression models.

	Model 1	Model 2	Model 3
	HR (95% CI)	HR (95% CI)	HR (95% CI)
**All‐cause mortality**			
Continuous	**1.52 (1.24, 1.87)**	**1.31 (1.09, 1.58)**	**1.39 (1.15, 1.68)**
Categories			
T1	Reference	Reference	Reference
T2	1.19 (0.81, 1.75)	1.10 (0.73, 1.66)	1.09 (0.72, 1.66)
T3	**1.94 (1.31, 2.85)**	1.29 (0.82, 2.03)	1.26 (0.79, 2.02)
P for trend	< 0.001	0.229	0.297
			
**Cardiovascular mortality**			
Continuous	**1.63 (1.27, 2.08)**	**1.43 (1.07, 1.91)**	**1.55 (1.11, 2.15)**
Categories			
T1	Reference	Reference	Reference
T2	2.26 (0.99, 5.17)	2.03 (0.93, 4.43)	2.16 (1.09, 4.30)
T3	**3.87 (1.82, 8.22)**	**2.41 (1.10, 5.28)**	**2.50 (1.22, 5.13)**
P for trend	< 0.0001	0.027	0.021

Model 1: unadjusted; Model 2: age, sex, and race; Model 3: further adjusted for BMI, income to poverty ratio, education level, smoking status, drinking status, hypertension, diabetes mellitus, and history of stroke.

**FIGURE 3:**
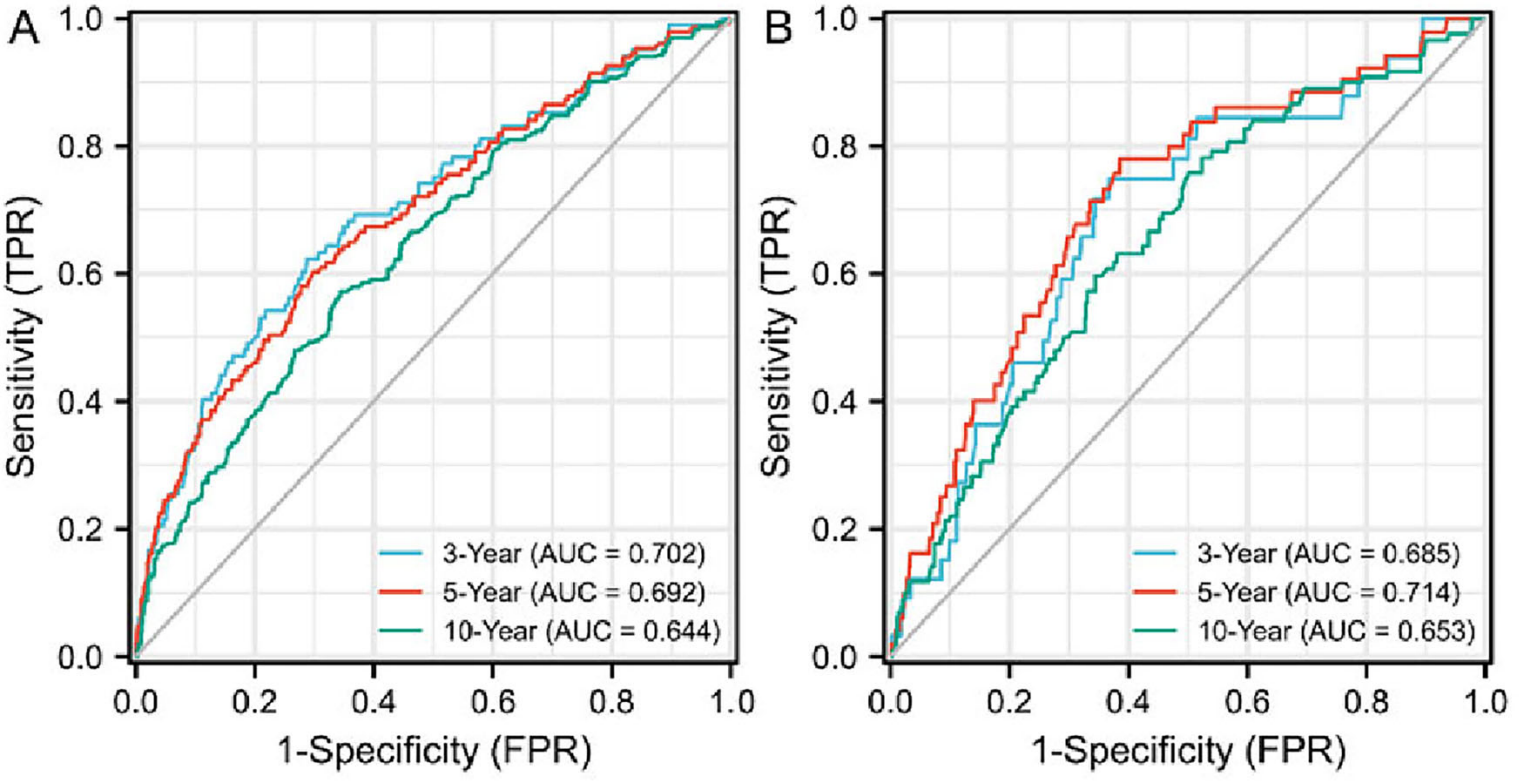
Predictive ability of SIRI for all‐cause mortality (A) and cardiovascular mortality (B).

The prognostic value of SIRI for all‐cause and cardiovascular mortality among patients with depression was evaluated using a time‐dependent ROC analysis. The results showed that the AUCs of SIRI were 0.702 (95% CI, 0.647–0.758), 0.692 (95% CI, 0.644–0.739), and 0.644 (95% CI, 0.603–0.685) for 3‐year, 5‐yea,r and 10‐year all‐cause mortality, respectively. The AUCs of SIRI were 0.685 (95% CI, 0.595–0.775), 0.714 (95% CI, 0.641–0.786), and 0.653 (95% CI, 0.586–0.720) for 3‐year, 5‐year, and 10‐year cardiovascular mortality, respectively (Figure 3, Table 3).

**TABLE 3:** Predictive ability of SIRI for all‐cause and CVD mortality.

	AUC	95% CI	Sensitivity	Specificity	Cut‐off point	Survival rate
**All‐cause mortality**						
3‐year	0.702	0.647–0.758	0.623	0.713	1.35	95.6%
5‐year	0.692	0.644–0.739	0.602	0.702	1.32	93.3%
10‐year	0.644	0.603–0.685	0.572	0.656	1.21	84.6%
**Cardiovascular mortality**						
3‐year	0.685	0.595–0.775	0.748	0.633	1.22	98.6%
5‐year	0.714	0.641–0.786	0.780	0.615	1.18	97.7%
10‐year	0.653	0.586–0.720	0.781	0.476	0.93	95.0%

In the subgroup analyses (Supplementary Figure S3), SIRI was generally positively associated with all‐cause and cardiovascular mortality in all subgroups, although the association was not statistically significant in some groups. There was a borderline significant association between cardiovascular mortality and age. Participants younger than 60 years were more vulnerable to higher SIRI, with an HR (95% CI) of 2.86 (1.82, 4.33). Interestingly, the association between SIRI and all‐cause mortality was not significant in participants aged < 60 years. A similar paradox was also found in stroke history. SIRI was significantly associated with increased cardiovascular mortality in participants with stroke history and increased all‐cause mortality in participants without stroke history.

Several sensitivity analyses revealed that the positive relationship between SIRI and all‐cause and cardiovascular mortality was robust after excluding participants who died within 2 years of follow‐up, further adjusting for the use of antihypertensive medication, and transforming SIRI into quartiles (Supplementary Tables S1–S3). We found that the positive association between SIRI and all‐cause and CVD mortality is robust among participants without depression. Interestingly, the HR for both all‐cause and CVD mortality is slightly higher in the non‐depressed population (Supplementary Table S4).

## 4 Discussion

This nationally representative sample study investigated the association between systemic inflammatory biomarker SIRI and survival outcomes among patients with depression. To the best of our knowledge, this is the first study to evaluate the role of SIRI in predicting all‐cause and cardiovascular mortality in patients with depression. In this nationally representative US cohort of 2410 participants from NHANES (2005–2018), we found that an increased SIRI was significantly associated with an increased risk of all‐cause and cardiovascular mortality after adjusting for confounding factors. These findings were consistent after sensitivity and subgroup analyses.

SIRI, comprising neutrophil, monocyte, and lymphocyte counts, is cost‐effective and commonly included in routine blood tests. Neutrophils and monocytes participate in the innate immune response, while lymphocytes are key to the adaptive or cell‐mediated immune response. SIRI, by integrating these immune pathways, captures the intricate interaction and systemic synergy between them, offering greater predictive power than individual neutrophil or lymphocyte measures. Research has shown a link between immune dysregulation and depression (Branchi et al. 2021; Miller and Raison 2016). They have also suggested that elevated SIRI levels in patients with depression may indicate an inflammatory burden of the disease (Li et al. 2023). In addition, increases in neutrophil/lymphocyte and monocyte/lymphocyte ratios have been reported to be associated with severity and cardiovascular risk factors in patients with depression (Aydin Sunbul et al. 2016; Marazziti et al. 2022; Özyurt and Binici 2018; Xia et al. 2023). These studies suggest that the combined indicator SIRI is a predictor of risk stratification in patients with depression and might be more objective in reflecting the interaction between the inflammatory and immune responses.

Inflammation results from immune system activation, involving immune responses along with vascular and molecular mediators. This can be triggered by various factors, including microbial infections, atherosclerosis, ischemia, and other internal and external influences. While numerous immune cells and mechanisms are essential for maintaining bodily homeostasis, their dysregulation can lead to disease. Elevated SIRI levels have been linked to increased all‐cause and cardiovascular mortality in hypertensive patients, as well as a heightened risk of sepsis and more severe strokes, according to previous studies (Jin et al. 2021; Zhang et al. 2021; Zhao et al. 2023). A meta‐analysis noted that approximately one‐quarter of patients with depression showed evidence of chronic low‐grade inflammation; notably, patients with major depression and elevated pro‐inflammatory biomarkers were less likely to respond to traditional antidepressive medications (Drevets et al. 2022; Osimo et al. 2019). This is consistent with the evidence that depression is associated with increased levels of pro‐inflammatory cytokines and acute‐phase proteins in blood and cerebrospinal fluid, altered adaptive immune responses, changes in the relative abundance of specific immune cell populations, and an increased propensity for autoimmune activation. In clinical trials, the immunosuppressive drugs, non‐steroidal anti‐inflammatory drugs (NSAIDs), and cytokine inhibitors, alone or in combination, produced better antidepressant effects (Bhatt et al. 2023).

We showed that a high SIRI correlates with increased all‐cause and cardiovascular mortality in depression, yet the precise mechanism by which an elevated SIRI raises these risks is still unclear. Research into the role of inflammation in mental illness is expanding, but it remains uncertain whether inflammation is a cause, consequence, or merely a correlate of depression (Bauer and Teixeira 2018; Franklin et al. 2018; Halaris 2019). Additionally, the observed link between inflammation and depression might be bidirectional or stem from shared underlying risk factors (Beurel et al. 2020; Cruz‐Pereira et al. 2020; Pitharouli et al. 2021).

Subgroup analysis by age showed that the association between SIRI and all‐cause mortality was not significant in participants aged < 60 years. One possible reason for this is the relatively small sample size of participants belonging to this group.

To our knowledge, this is the first study assessing SIRI's predictive value for all‐cause and cardiovascular mortality in patients with depression. Using a national sample, we demonstrated that SIRI, an innovative inflammatory biomarker, could predict mortality risks in depressed individuals. Adjustments for covariates and multiple sensitivity analyses confirmed the robustness of our findings.

However, our study has limitations. Primarily, depression was not clinically diagnosed but identified via PHQ‐9 scores, a tool considered effective for screening depressive symptoms. Additionally, the number of outcome events, particularly cardiovascular deaths, was limited, and our findings therefore require validation in larger studies. SIRI calculations were based on a single whole blood measurement, potentially influenced by the inflammatory status at the time of testing; to reduce bias, we excluded participants with abnormal blood cell counts. Lastly, despite adjusting for known risk factors, some covariates might have been overlooked.

## 5 Conclusion

In conclusion, we analyzed 2410 participants with depression from the 2005–2018 NHANES database and revealed positive associations of SIRI with the risk of all‐cause and cardiovascular mortality during long‐term follow‐up. Our findings suggest that SIRI may be a useful biomarker for risk stratification of all‐cause and cardiovascular mortality in patients with depression. Further studies are needed to validate its clinical utility.

## Author Contributions

**Ying Tang**: conceptualization, formal analysis, writing – original draft, writing – review and editing. **Songfeng Zhao**: conceptualization, formal analysis, writing – original draft, writing – review and editing. **Qiuping Huang**: investigation and data curation. **Xinxin Chen**: investigation and data curation. **Shuhong Lin**: investigation and data curation. **Yifan Li**: validation and visualization. **Jingyue Hao**: validation and resources. **Zhenjiang Liao**: conceptualization, methodology, and supervision. **Hongxian Shen**: conceptualization, funding acquisition, supervision, writing – review and editing.

## Funding

The authors have nothing to report.

## Ethics Statement

This study was conducted according to the guidelines laid down in the Declaration of Helsinki, and all procedures involving research study participants were approved by the institutional review board of the National Center for Health Statistics (NCHS).

## Consent

Written informed consent was obtained from all participants/patients.

## Conflicts of Interest

The authors declare no conflicts of interest.

## Supporting information




**Supplementary Materials**: brb371437‐sup‐0001‐tablesS1‐S4.docx

**Supplementary Materials**: Figure S1. Flow chart of participant enrollment.

**Supplementary Materials**: Figure S2. Association between depression severity and SIRI levels.

**Supplementary Materials**: Figure S3. Subgroup analysis of the association between SIRI and all‐cause mortality (A) and cardiovascular mortality (B) among depressive participants.

## Data Availability

Publicly available datasets were analyzed in this study. This data can be found here: https://www.cdc.gov/nchs/nhanes/.
