# Supplementary material for: Association of Systemic Inflammation Response Index With All‐Cause and Cardiovascular Mortality Among Individuals With Depression: NHANES 2005–2018
Source: Brain Behav. 2026 Apr 22;16(4):e71437. doi: 10.1002/brb3.71437 (PMC13103469; doi:10.1002/brb3.71437)
Supplement: Supplementary file 1 — Supplementary Materials: brb371437‐sup‐0001‐tablesS1‐S4.docx [file BRB3-16-e71437-s001.docx]

Table S1. Risk of all-cause and CVD mortality among depressive participants according to SIRI after exclusion died within 2 years of follow-up.

|  | Model 1 | Model 2 | Model 3 |
| --- | --- | --- | --- |
|  | HR (95% CI) | HR (95% CI) | HR (95% CI) |
| **All-cause mortality** |  |  |  |
| Continuous | **1.42 (1.14, 1.77)** | **1.23 (1.00, 1.52)** | **1.33 (1.07, 1.65)** |
| Categories |  |  |  |
| T1 | Reference | Reference | Reference |
| T2 | 1.27 (0.86, 1.86) | 1.21 (0.80, 1.82) | 1.21 (0.80, 1.81) |
| T3 | **1.82 (1.24, 2.68)** | 1.29 (0.81, 2.05) | 1.28 (0.80, 2.06) |
| P for trend | 0.002 | 0.323 | 0.349 |
|  |  |  |  |
| **CVD mortality** |  |  |  |
| Continuous | **1.57 (1.16, 2.12)** | **1.42 (1.00, 2.01)** | **1.58 (1.06, 2.36)** |
| Categories |  |  |  |
| T1 | Reference | Reference | Reference |
| T2 | 2.56 (0.93, 7.02) | 2.39 (0.91, 6.28) | **2.68 (1.19, 6.02)** |
| T3 | **3.74 (1.43, 9.80)** | 2.53 (0.97, 6.61) | **2.85 (1.23, 6.60)** |
| P for trend | 0.030 | 0.063 | 0.035 |

Model 1: unadjusted. Model 2: age, sex, race Model 3: further adjusted for BMI, income to poverty ratio, education level, smoking status, drinking status, hypertension, diabetes mellitus, and history of stroke.

Table S2. Risk of all-cause and CVD mortality among depressive participants according to SIRI.

|  | Model 4 |
| --- | --- |
|  | HR (95% CI) |
| **All-cause mortality** |  |
| Continuous | **1.38 (1.16, 1.68)** |
| Categories |  |
| T1 | Reference |
| T2 | 1.10 (0.73, 1.67) |
| T3 | 1.26 (0.79, 2.03) |
| P for trend | 0.293 |
|  |  |
| **CVD mortality** |  |
| Continuous | **1.50 (1.12, 2.14)** |
| Categories |  |
| T1 | Reference |
| T2 | **2.16 (1.10, 4.32)** |
| T3 | **2.41 (1.23, 5.15)** |
| P for trend | 0.021 |

Model 4: adjusted for age, sex, race, BMI, income to poverty ratio, education level, smoking status, drinking status, hypertension, diabetes mellitus, and history of stroke.

Table S3. Risk of all-cause mortality among depressive participants according to quartiles of SIRI

|  | Model 1 | Model 2 | Model 3 |
| --- | --- | --- | --- |
|  | HR (95% CI) | HR (95% CI) | HR (95% CI) |
| **All-cause mortality** |  |  |  |
| T1 | Reference | Reference | Reference |
| T2 | 1.42 (0.85, 2.37) | 1.33 (0.81, 2.16) | 1.25 (0.78, 2.11) |
| T3 | **1.67 (1.03, 2.70)** | **1.44 (0.86, 2.41)** | 1.34 (0.80, 2.30) |
| T4 | **2.59 (1.65, 4.05)** | **1.56 (0.95, 2.58)** | 1.48 (0.90, 2.51) |
| P for trend | <0.001 | 0.127 | 0.167 |
| **CVD mortality** |  |  |  |
| T1 | Reference | Reference | Reference |
| T2 | 1.03 (0.37, 2.88) | 0.92 (0.35, 2.43) | 0.84 (0.39, 2.19) |
| T3 | **2.46 (1.04, 5.84)** | 2.07 (0.93, 4.59) | **1.96 (1.06, 4.16)** |
| T4 | **3.31 (1.44, 7.60)** | 1.84 (0.75, 4.50) | 1.69 (0.83, 4.22) |
| P for trend | <0.001 | 0.087 | 0.079 |

Model 1: unadjusted. Model 2: age, sex, race Model 3: further adjusted for BMI, income to poverty ratio, education level, smoking status, drinking status, hypertension, diabetes mellitus, and history of stroke.

Table S4. Risk of all-cause and cardiovascular mortality among participants without depression.

|  | Model 1 | Model 2 | Model 3 |
| --- | --- | --- | --- |
|  | HR (95% CI) | HR (95% CI) | HR (95% CI) |
| **All-cause mortality** |  |  |  |
| Continuous | **1.72 (1.63, 1.81)** | **1.37 (1.30, 1.43)** | **1.32 (1.26, 1.39)** |
| Categories |  |  |  |
| T1 | Reference | Reference | Reference |
| T2 | **1.34 (1.15, 1.55)** | 1.09 (0.94, 1.26) | 1.07 (0.91, 1.25) |
| T3 | **2.95 (2.60, 3.34)** | **1.60 (1.40, 1.81)** | 1.45 (1.25, 1.69) |
| P for trend | <0.001 | <0.001 | <0.001 |
|  |  |  |  |
| **Cardiovascular mortality** |  |  |  |
| Continuous | **1.25 (1.15, 1.36)** | **1.22 (1.14, 1.31)** | **1.28 (1.10, 1.28)** |
| Categories |  |  |  |
| T1 | Reference | Reference | Reference |
| T2 | 1.20 (0.94, 1.52) | 1.12 (0.90, 1.38) | 1.10 (0.88, 1.38) |
| T3 | **1.67 (1.37, 2.04)** | **1.42 (1.17, 1.73)** | **1.44 (1.13, 1.82)** |
| P for trend | <0.001 | <0.001 | <0.001 |

Model 1: unadjusted; Model 2: age, sex, and race; Model 3: further adjusted for BMI, income to poverty ratio, education level, smoking status, drinking status, hypertension, diabetes mellitus, and history of stroke.
