# Supplementary figures and images for: Association of Systemic Inflammation Response Index With All‐Cause and Cardiovascular Mortality Among Individuals With Depression: NHANES 2005–2018
Source: Brain Behav. 2026 Apr 22;16(4):e71437. doi: 10.1002/brb3.71437 (PMC13103469; doi:10.1002/brb3.71437)

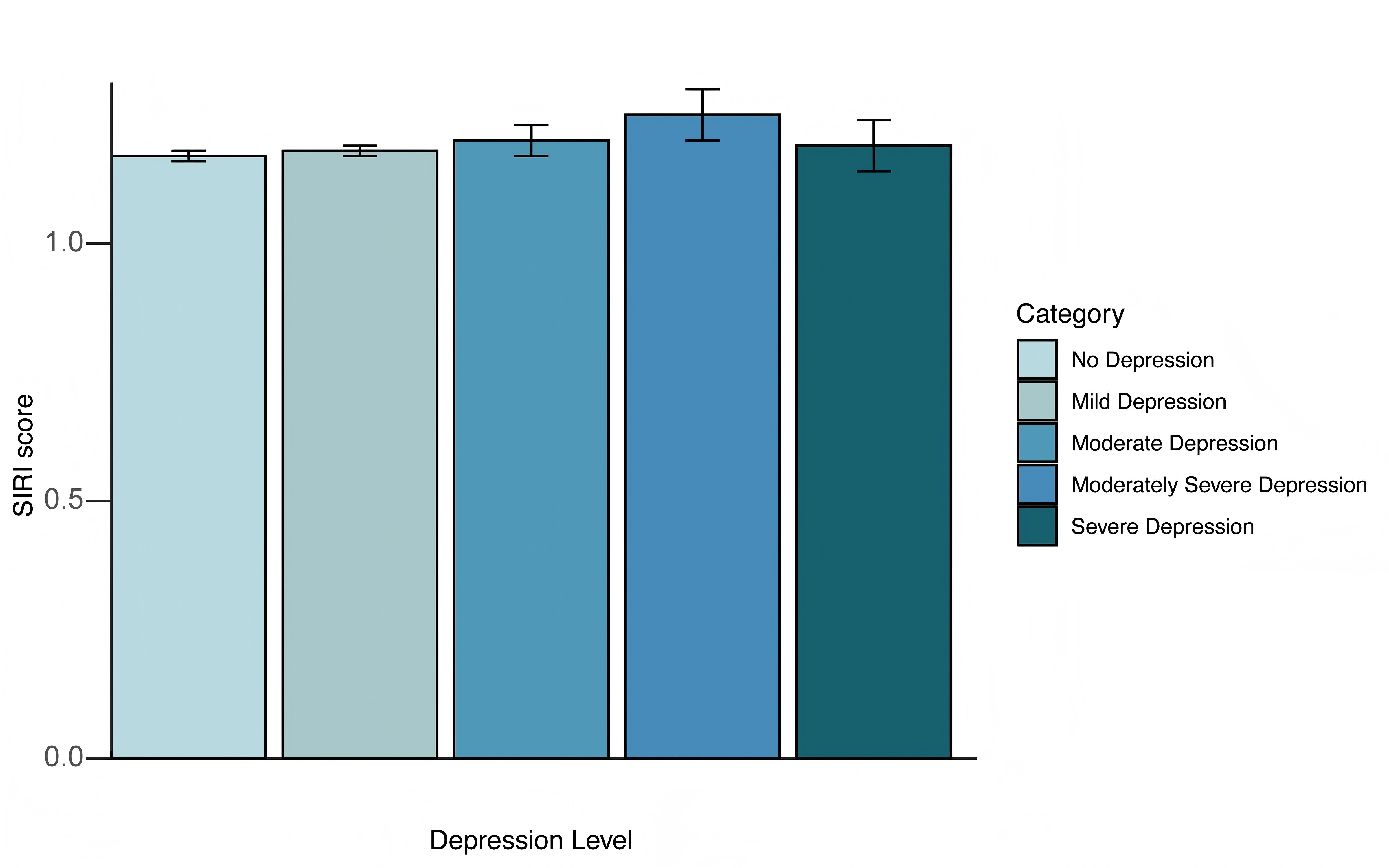

Supplement: Supplementary file 3 — Supplementary Materials: Figure S2. Association between depression severity and SIRI levels. [file BRB3-16-e71437-s004.bmp]

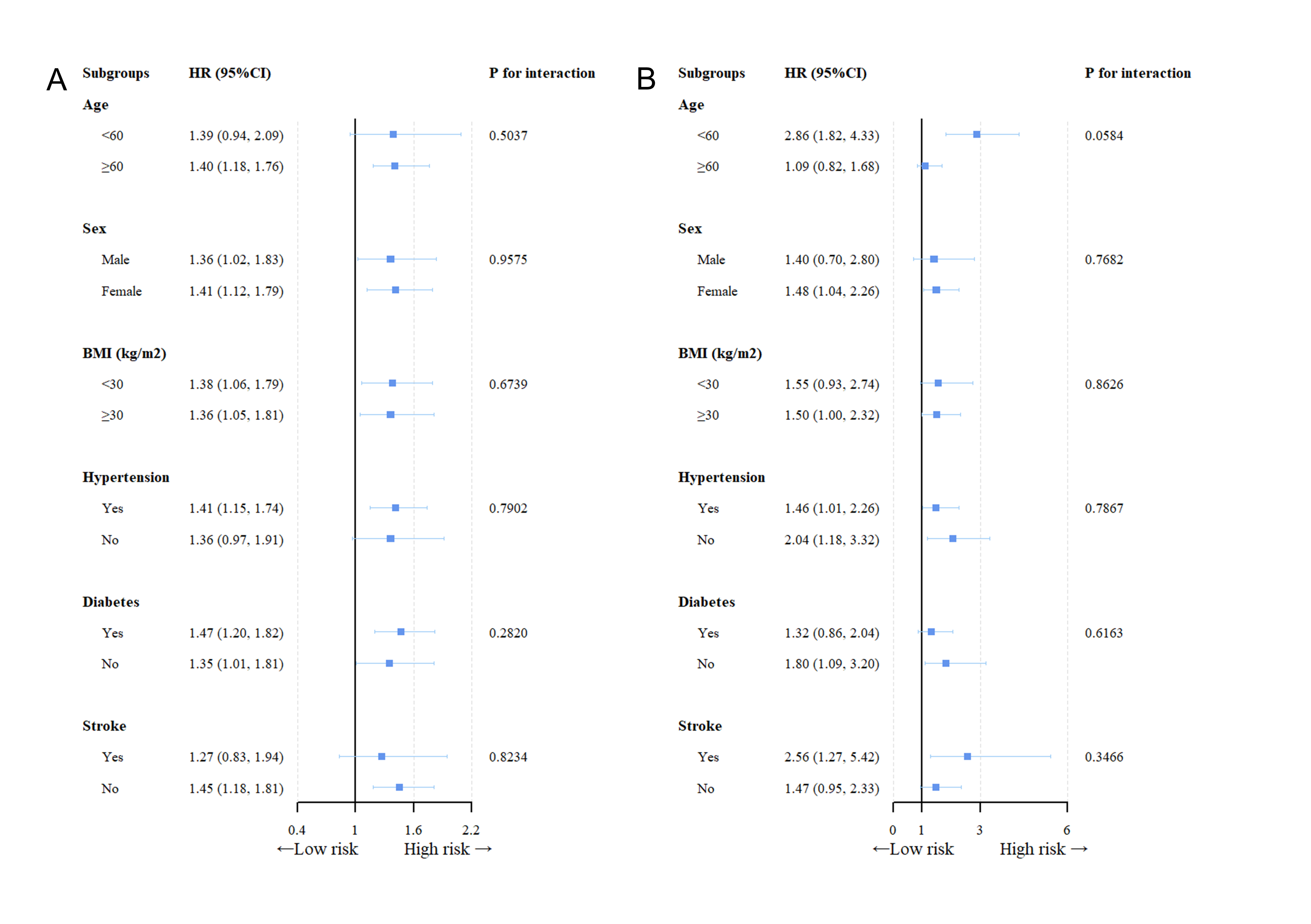

Supplement: Supplementary file 4 — Supplementary Materials: Figure S3. Subgroup analysis of the association between SIRI and all‐cause mortality (A) and cardiovascular mortality (B) among depressive participants. [file BRB3-16-e71437-s003.bmp]
